# Evaluation of cellular response and drug delivery efficacy of nanoporous stainless steel material

**DOI:** 10.1186/s40824-021-00232-8

**Published:** 2021-09-26

**Authors:** Inho Bae, Kyung-Seob Lim, Jun-Kyu Park, Ju Han Song, Sin-Hye Oh, Jung-Woo Kim, Zijiao Zhang, Chan Park, Jeong-Tae Koh

**Affiliations:** 1 Hard-tissue Biointerface Research Center; Department of Pharmacology and Dental Therapeutics, School of Dentistry, Chonnam National University, Gwangju, 61186 Republic of Korea; 2 National Primate Research Center & Futuristic Animal Resource and Research Center, Korea Research Institute of Bioscience and Biotechnology, Ochang, 28116 Republic of Korea; 3 Department of Polymer Science and Engineering, Sunchon National University, Suncheon, 57922 Republic of Korea; 4 Department of Prosthodontics, School of Dentistry, Chonnam National University, Gwangju, 61186 Republic of Korea

**Keywords:** Nanoporous structure, Stainless steel, Surface modification, Cellular response, Drug delivery

## Abstract

**Objective:**

Various surface modification techniques that can further improve the function and usability of stainless steel as a medical device have been reported. In the present study, the physical and biological properties of nanoporous stainless steel as well as its usefulness for drug delivery were assessed.

**Methods:**

The specimen was prepared with a circular disk shape (15 mm in diameter and 1 mm in thickness). The disk was subjected to electropolishing at a constant voltage of 20 V and 10 A for 10 min in an acidic environment (50% H_2_SO_4_). Everolimus (EVL) was used as a testing drug for drug-loading capacity of the material surface and release kinetics. The physiobiological properties of the material were assessed using platelet adhesion, and smooth muscle cell (SMC) adhesion, migration, and proliferation assays.

**Results:**

The surface roughness of the postpolishing group was greater than that of the nonpolishing group. Platelet adhesion and SMC adhesion and migration were inhibited in the postpolishing group compared to those in the prepolishing group. In the postpolishing group, the total amount of EVL on the surface (i.e., drug storage rate) was higher and the drug release rate was lower, with half the amount of the EVL released within 4 days compared with only 1 day for that of the prepolishing group.

**Conclusion:**

Taken together, this stainless steel with a nanoporous surface could be used as a medical device for controlling cellular responses and carrying drugs.

## Introduction

Various surface modification techniques that improve the function and usability of stainless steel as a medical device have been reported. Among these, the porous surface of stainless steel may not only improve biocompatibility [1], but also provide an environment in which biological agents and drugs can be efficiently transported. These innovative methods help in creating specific surfaces that allow binding of the target drug onto the metal surface. Thus, biophysical surface modification and optimization have gained immense interest in research of biomaterial sciences [2, 3]. Moreover, such surface modification methods have recently been used to improve the blood [4] and tissue compatibility of biomaterials [5]. All implantable biomaterials are directly exposed to the local environment [6]. Thus, implantable metal-related thrombosis and inflammation are the main issues that affect the surface properties [7, 8]. Several studies have reported that the formation of nanopores on an implant surface improves its biocompatibility [9, 10]. In addition, certain studies have reported the efficacy of drugs delivered through titanium-based nanotubes [11, 12]. Despite the advantages of nanoporous surfaces, little information is available on nanoporous stainless steel (316 L). Previously, we incorporated a protein into nanoscale pores on the surfaces of medical devices by a simple loading process [9]. The metals used for medical devices, including stainless steel, have a native oxide layer on the surface that forms spontaneously on the top few nanometers of the bulk material and provides the required resistance to corrosion. Previous studies have reported that the surface layer modifications reveal the importance of the oxide layer for biological reactions [13]. Although most of these properties are determined by the composition of the bulk material, biological reactions can be controlled by surface modifications. Previously, we have reported that the formation of micropores on the surface of medical devices can have a positive effect on cells [14]. Furthermore, it has been reported that surface modification via micropore and patterning can improve the function of the metal as an implanted material by controlling the amount of drug in it [15].

Everolimus (EVL), a derivative of sirolimus, which inhibits the mammalian target of rapamycin (mTOR) protein, is generally coated on the coronary stent. As this drug can reduce restenosis, it has been widely studied and used in patients with coronary artery diseases [16, 17]. Stainless steel is frequently used in biomedical applications, such as orthopedic implants, owing to its properties such as high corrosion and fatigue resistance as well as high fracture toughness. In addition to biocompatibility, these properties are important in the selection and adaptation of a material for biomedical applications. The aim of the present study was to fabricate a stainless steel with a nanoporous surface and to evaluate its physiobiological properties. Additionally, the drug storage and delivery capacities of the porous stainless steel were evaluated to verify its feasibility for coronary stent and orthopedic use.

## Materials and methods

### Sample preparation

**Fig. 1:**
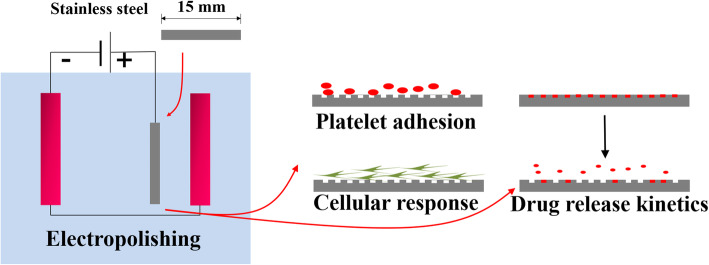
A schematic illustration of the experimental setup

Stainless steel was used for this study in a circular disk shape (316 L; 15 mm in diameter and 1 mm in thickness). The metal disk was first processed for 1 h in an acidic environment (50% H_2_SO_4_) to remove and crush the burr. Thereafter, the samples underwent electropolishing (E-polishing), as described in our previous reports [18, 19]. In brief, the disks were positioned lengthwise in the E-polishing chamber containing H_2_SO_4_. The temperature of the electrolyte solution was maintained at 50 °C. E-polishing was performed at a constant voltage of 10 V and 10 A for 10 min, using a DC power supply (Fine Power F-3005; SG EMD, Anyang, Korea). The anodizing electrolyte comprised 1 M H_2_SO_4_ and 1.0 wt% hydrofluoric acid solutions with a pH of 2–3. A platinum plate was used as the anode, and the distance between the anode and cathode was 10 mm. All anodic oxidation processes were carried out at room temperature. After oxidation, the specimens were washed with water for 20 min and then dried for 24 h in an oven at 37 °C (Fig. 1).

### Morphological examination of the material surface

The morphologies of the metal surface and the cells adhered to it were examined via scanning electron microscopy (SEM; Hitachi, Tokyo, Japan) at voltages ranging from 5 to 15 kV after sputter coating the surfaces with platinum.

### Investigation of surface properties

The metal surface properties were assessed via atomic force microscopy (AFM; Nano Scope IIIa, Digital Instruments, USA). The measured surface roughness was presented as a roughness average (Ra) of three tests. The Ra value for each specimen was defined as the arithmetic average of all absolute distances of the roughness profile from the center line within the measuring length.

### Examination of platelet adhesion on the metal surface

To estimate the thrombogenicity of the E-polished metal surface, the platelet adhesion test (a type of hemocompatibility test) was performed [20]. Platelet-rich plasma (PRP) was obtained by centrifuging (150×*g* for 15 min and 500×*g* for 20 min) the fresh porcine whole blood with 5 mL of a 3.0 wt% sodium citrate solution. The PRP was harvested carefully and diluted to 3.0 × 10^7^ platelets/mL. Next, the PRP solution was loaded onto the metal surfaces and allowed to instill at room temperature for 180 min. Thereafter, the PRP that was adhered nonspecifically to the surface was removed by gentle rinsing. Prior to SEM observation, the samples were dehydrated with a gradient concentration of ethanol and then air dried. The platelet count was enumerated by manual counting.

### Morphological analysis of adhered cell

For observing cell adherence, the vascular smooth muscle cell (SMC) isolated from aortic media of male rats [21] was seeded onto pre- and postpolished disks, respectively, at a density of 5 × 10^4^ cells/cm^2^. After 6 h of cultivation, the cell-seeded disks were subjected to SEM. Prior to imaging, the surfaces were rinsed twice in phosphate-buffered saline (PBS) and then soaked in the primary fixative of 2.5% glutaraldehyde. Thereafter, the surfaces were washed twice for 5 min with PBS buffer. Next, the cells were dehydrated by replacing the buffer with increasing concentrations of ethanol (40 ~ 100%) for 10 min at each concentration. Subsequently, the cells were air dried at room temperature. The cell count was enumerated by manual counting.

### Everolimus loading

As we previously reported, 10 mg/ml of EVL, which is the optimal drug concentration for in vitro experiments [15], was dissolved in tetrahydrofuran (THF) and 100 μL of EVL solution was loaded onto the specimen surface. Thereafter, the drug was gently shaken so that the drug was uniformly coated for 24 h. coated evenly for 24 h. After gently washing the surface with de-ionized water to remove simple laid EVL from the surface, the plates were lyophilized.

### Quantification of the drug amount and release rate

The total amount of EVL on the disk surfaces pre- and post-E-polishing were estimated. In brief, 100 μL of EVL solution (10 mg/mL) was carefully loaded onto the disk surface and incubated for 24 h to allow drug instillation. Thereafter, after gentle washing of the surface with deionized water to remove the noninstilled EVL, the disk was lyophilized as described previously [22, 23]. To measure the total amount of instilled EVL, the disk was immersed in *tetrahydrofuran* (THF) solution with gentle shaking. The THF solution was then analyzed using an ultraviolet-visible (UV-*vis*) spectrophotometer (Multiskan EX; ThermoFisher Scientific, Waltham, MA, USA) at 278 nm. The readings were continuously noted until the UV value was zero. The cumulative values were set as 100%. To determine the release rate of EVL from the disk, the lyophilized disk was placed in PBS (pH 7.2) solution, and samples of the solution were withdrawn on designated days for measuring the absorbance at 270 nm with the UV-*vis* spectrophotometer. The concentration of drug released was calculated by comparison against the drug standard curve and was expressed as a cumulative amount relative to the total amount of EVL on the disk.

### Cell migration assay

SMC (1 × 10^4^ cells/cm^2^) was seeded into 24-well cell culture dishes. After 24 h of incubation, a line of 50 μm width was created by scraping through the center of the cell monolayer with a sterile tip. To examine the indirect cellular response of EVL released from the disks, the EVL-instilled pre- or postpolished disk was washed with PBS and then positioned onto a Transwell membrane insert (8.0 μm pore size; Corning Inc., Corning, NY, USA). The Transwell insert was then assembled into 24-well cell culture dishes that had been preseeded with SMC 1 day prior. After 24 h of additional incubation at 37 °C in a humidified 5% CO_2_ atmosphere, fields of the scratched areas were randomly selected for imaging [19]. Cell migration was calculated as [(initial scratched area − remaining scratched area at 24 h incubation)/initial scratched area] × 100.

### In vitro cell proliferation assay

To verify the effect of E-polishing on SMC proliferation of EVL released from the disk, SMC (1 × 10^4^ cells/cm^2^) was first seeded into 24-well cell culture dishes and incubated at 37 °C in a humidified 5% CO_2_ atmosphere. After 1 d of cultivation, the EVL released from the disk was added to the 24-well culture dish. Cell proliferation on the disks was examined via XTT (2,3-*bis*-(2-methoxy-4-nitro-5-sulfophenyl)-2*H*-tetrazolium-5-carboxanilide) assay after 1, 2, 4, and 7 d of cultivation, using an EZ-Cytox cell viability assay kit (Daeil Lab Service Co., Seoul, Korea).

### Statistical analysis

Statistical analysis was performed using the commercially available software SPSS version 15 (SPSS, Chicago, IL, US). Data are presented as the mean ± SD. The unpaired Student’s *t* test was used for comparing data of different groups. Values of *p* < 0.05 were considered statistically significant.

## Results

### Surface morphology of the metal surfaces

**Fig. 2:**
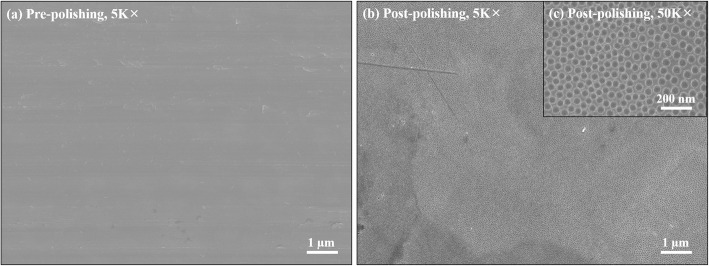
Morphological analysis of the disk surfaces. Representative SEM images of prepolishing (**a**) and postpolishing materials (**b**). (**c**) Magnification of (**b**)

**Fig. 3:**
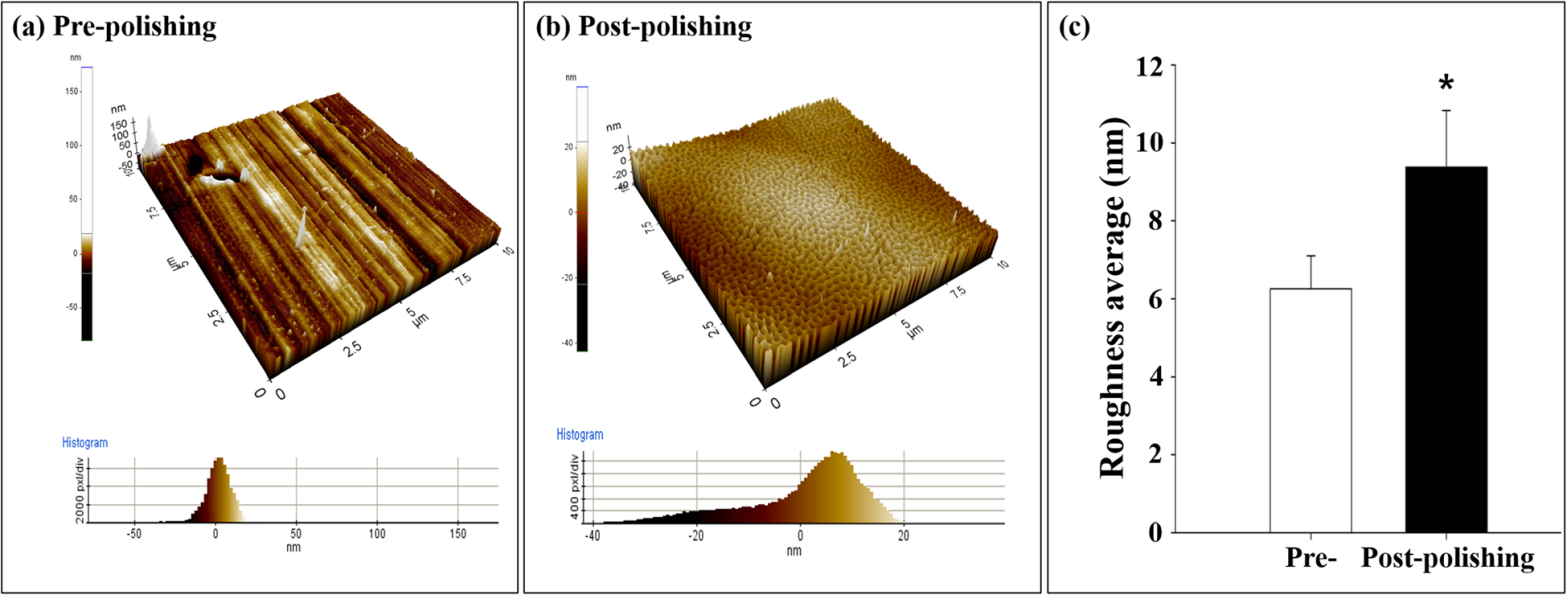
Characterization of the material surfaces by AFM. Representative images of prepolishing (**a**) and postpolishing surfaces (**b**). **c** Quantitative analysis of the AFM images. The indicated values represent the mean ± SD (*n* = 5), *p < 0.05

The surface morphology and roughness of the metal surfaces pre- and post-E-polishing were examined via SEM. A round shaped disk (15 mm in diameter) was utilized for this study. The sample that did not undergo E-polishing process revealed a smooth surface, with no unusual shapes (Fig. 2a). In contrast, the E-polished surface had nanopores of approximately 100 nm in diameter (Fig. 2b, c). The variation in the surface-modified roughness was analyzed via AFM topography. The three-dimensional AFM images revealed that the Ra was increased (53.3%) in the postpolishing group compared with that in the prepolishing group (Ra: prepolishing 6.1 ± 1.02 nm; postpolishing 9.2 ± 1.74 nm, *n* = 5, *p* < 0.05; Fig. 3).

### Platelet adhesion on the surfaces

**Fig. 4:**
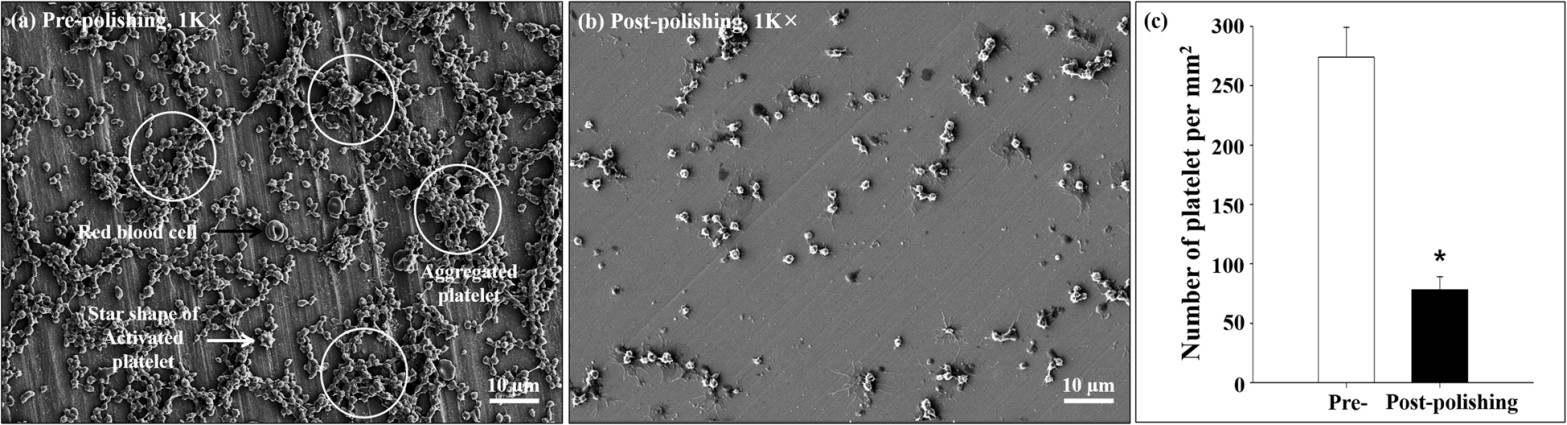
Platelet adhesion assay. Representative images of platelets on prepolishing (**a**) and postpolishing surfaces (**b**). **c** Quantitative analysis of the images. White circles: aggregated platelets; black arrow: red blood cells; white arrow: star-shaped activated platelets. The indicated values represent the mean ± SD (*n* = 5), *p < 0.05

The morphology and distribution of platelets on the surface were assessed by calculating the number and size of blood platelets. Numerous platelets were aggregated, star-shaped, and interconnected with each other in the prepolishing group (Fig. 4a). In contrast, only few connected blood platelets appeared in the postpolishing group (Fig. 4b). The number of blood platelets in the postpolishing group (78 ± 11/mm^2^) was markedly less (71.5%) than that in the prepolishing group (274 ± 26/mm^2^, *n* = 5, *p* < 0.05; Fig. 4c) under the same conditions.

### Cell adhesion and morphology

**Fig. 5:**
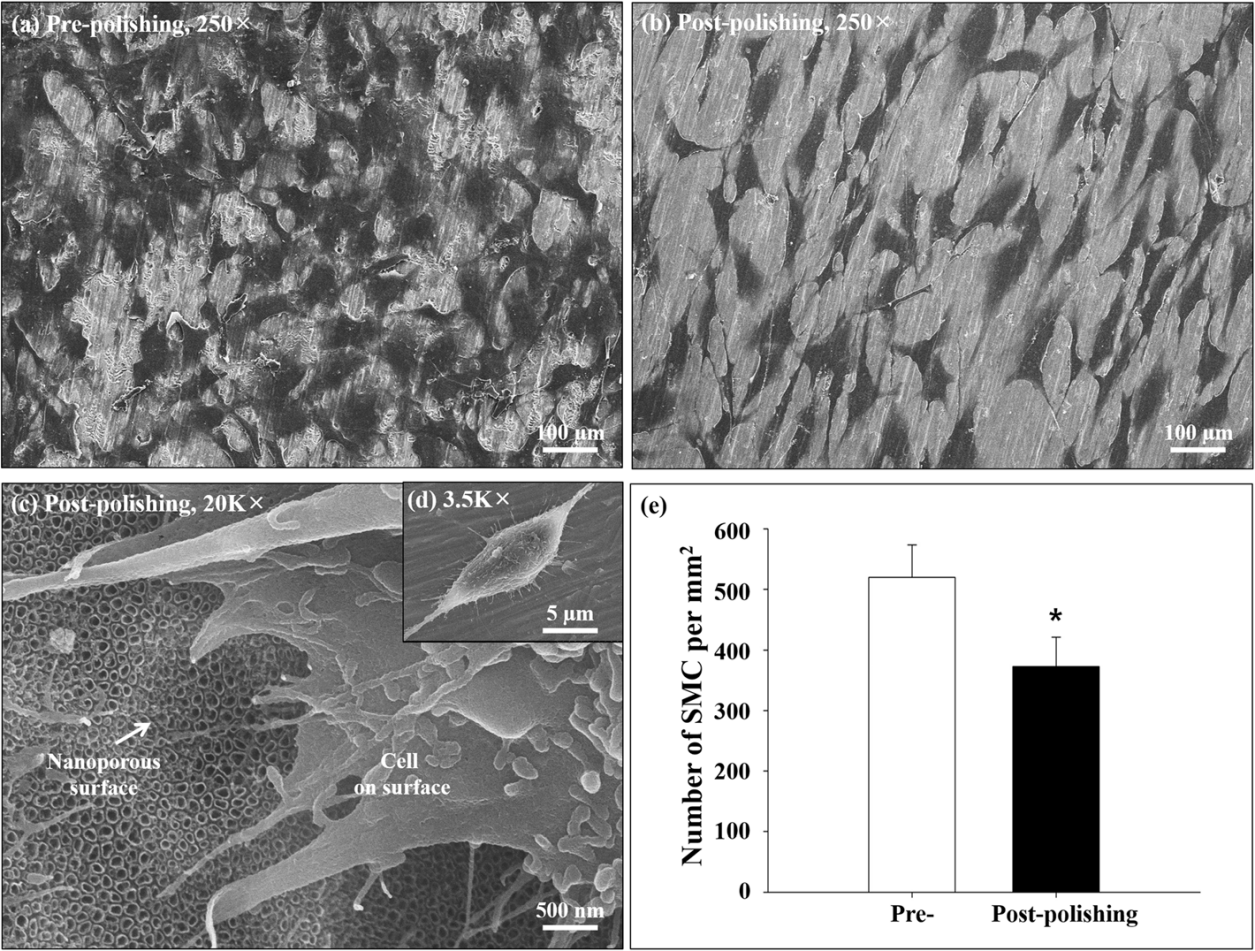
Smooth muscle cell adhesion. Representative images of prepolishing (**a**) and postpolishing surfaces (**b**, **c**, and **d**). **e** Quantitative analysis of the number of adhered smooth muscle cells. The indicated values represent the mean ± SD (*n* = 5), *p < 0.05

The SMC onto the surface was adhered well in the prepolishing group (Fig. 5a); whereas, it was spread flat, robust, and shaped like filopodia in the postpolishing group (Fig. 5b, c, and d). The SMC count was decreased (28.2%) in the postpolishing group (373.0 ± 48.43/mm^2^) compared to that in the prepolishing group (519.8 ± 53.57/mm^2^, *n* = 5, *p* < 0.05; Fig. 5e).

### Drug release rate

**Fig. 6:**
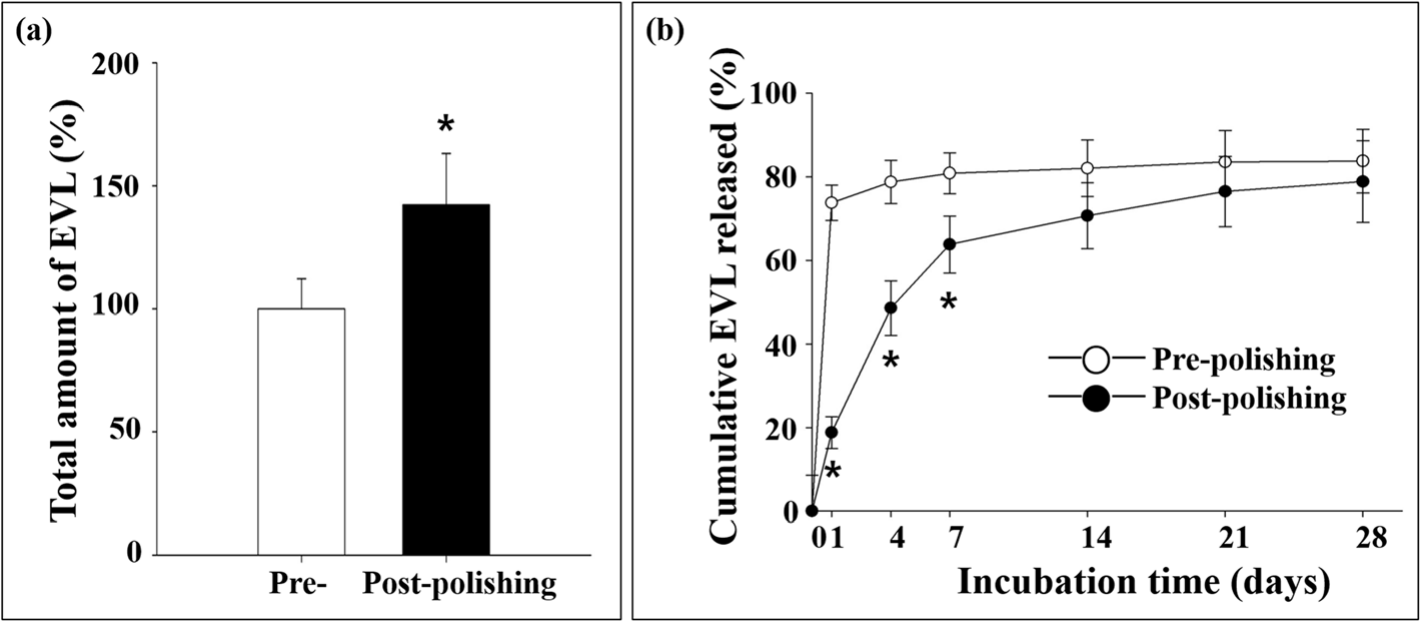
Total everolimus amount and its release kinetics from the disk surfaces. **a** Total amount of EVL on the surfaces. **b** Cumulative EVL released from the surfaces. The indicated values represent the mean ± SD (*n* = 5), *p < 0.05

The EVL release rate from the material surface was examined, as described in the Materials and methods section. The point peak shift in PBS (calculated at 270 nm) slightly differed from that in THF. The total amount of EVL released in the postpolishing group (142.3% ± 10.63%) was higher than that in the prepolishing group (99.2% ± 7.96%, *n* = 5, *p* < 0.05; Fig. 6a). Each value was set as the total amount, which was then used to compare the measured amount of EVL released from the disk. As a result, the drug release rate was slower for the postpolishing group, where half the amount of EVL was released within 4 days as compared with only 1 day for the prepolishing group (Fig. 6b).

### Cell migration and proliferation assay

**Fig. 7:**
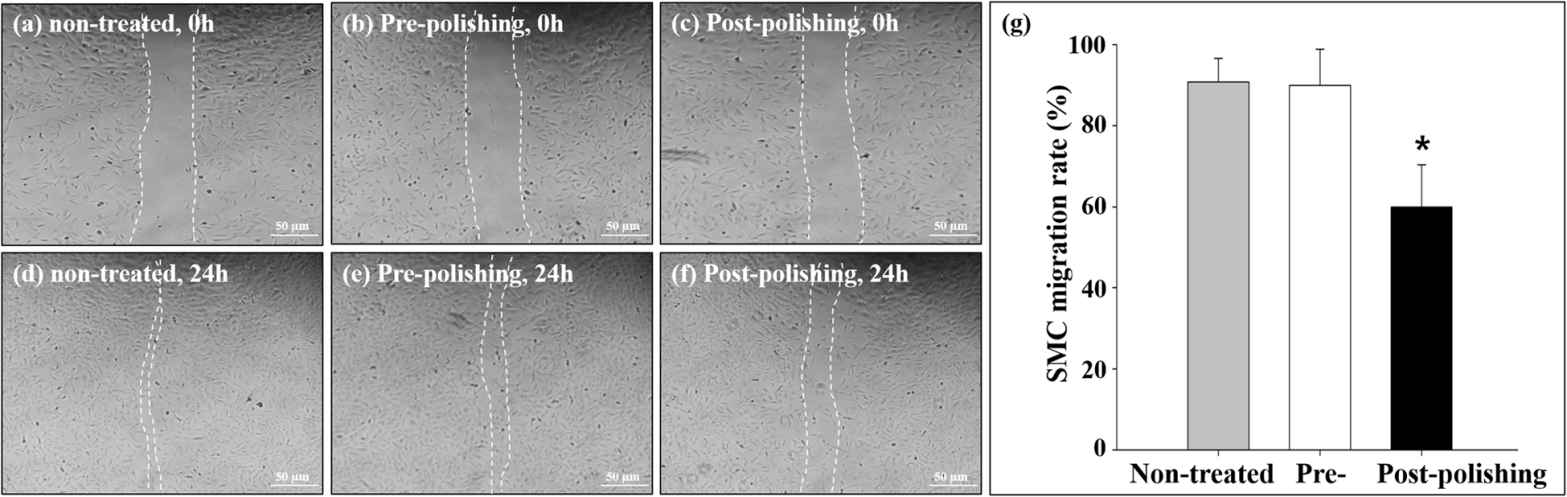
Smooth muscle cell migration. Representative images of cell migration from 0 to 24 h of postscratch cultivation. Nontreated (**a**, **d**), prepolishing (**b**, **e**), and postpolishing surfaces (**c**, **f**) are depicted. **g** Image analysis of the cell migration rate, calculated as [(initial scratch area – remaining scratch area during 24-h cultivation)/initial scratch area] × 100. The indicated values are the mean ± SD (*n* = 5), *p < 0.05

**Fig. 8:**
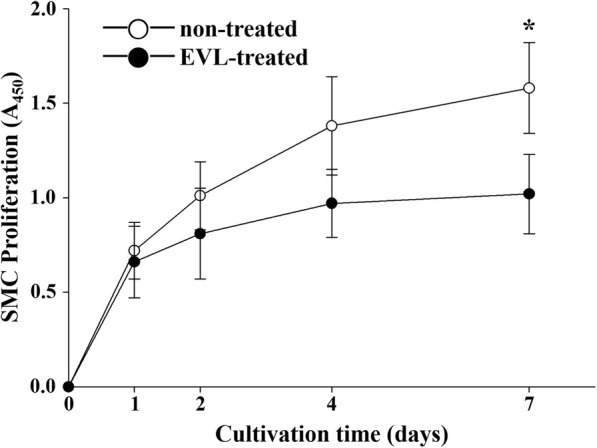
XTT assay of the inhibitory effect of everolimus released from the material surfaces, on smooth muscle cell proliferation. The indicated values represent the mean ± SD (*n* = 5); *p < 0.05 compared with the nontreated control at the same time point

As described in materials and methods section, the middle region of the cell monolayer on the culture dish was scratched (12.0 ± 1.05 mm^2^; Fig. 7a, b, and c). After 24 h of cultivation, the SMC migration rate in the prepolishing group (1.2 ± 1.31 mm^2^, 90.0 ± 8.86%; Fig. 7e) was similar to that in the nontreated group (1.1 ± 3.31 mm^2^, 90.8 ± 5.84%; *n* = 5, *p* = NS; Fig. 7d). In contrast, the SMC migration rate was decreased in the postpolishing group (4.8 ± 5.04 mm^2^, 60.0% ± 10.28%, *n* = 5, *p* < 0.05; Fig. 7f), where it was approximately 33.9% slower than that in the prepolishing group (Fig. 7g). To investigate the retention of EVL function after its release from the disk, its inhibitory effect on the proliferation of SMC, which is a component of the tunica media of the vascular wall [24], was evaluated. The proliferation of SMC was remarkably inhibited in the EVL-treated group (1.0 ± 0.21 at 7 days, 45.1% ± 4.25%) than that in the control group (1.6 ± 0.24, *n* = 5, *p* < 0.05; Fig. 8).

## Discussion

In the last decade, stainless steel was widely used in developing medical devices, particularly coronary stent, dental posts, fixtures, screws, and orthopedic implants. Previous studies have reported various methods to improve performance and function of stainless steel through surface modification such as formation of microstructure and porous layer [25, 26]; however, researches on the cellular response and drug delivery efficacy of the surface-modified stainless steel material through the formation of nanoporous are limited. Previously, we had reported that nanopores on metal surfaces are able to store and deliver drugs to the targeted region [9]. Drug anchoring by physical methods (e.g., electrospinning, negative pressure, and lyophilization) enable the exclusion of polymers during drug-eluting stent fabrication. Of course, it has been reported that the thickness of the stent strut affects various pathological outcomes [27]. However, in this study, only the surface properties of the material were studied. Through follow-up research, it will be necessary to conduct a study on the thickness of the strut using a 3-dimensional stent.

In the present study, E-polishing was employed as a surface modification method to form nanopores on the stainless steel surface. The SEM findings revealed the remarkable creation of uniform nanopores of approximately 100 nm in diameter on the metal surface after E-polishing (Fig. 2). Moreover, the AFM images revealed a compact layer with a columnar structure. The surface roughness of the prepolishing group was relatively smooth, whereas the Ra value was increased (Fig. 3) in the postpolishing group. These results indicate that the polishing process altered the surface topology. Medical devices, which constitute a foreign particle, may lead to thrombosis events. Therefore, the first interaction between the blood and the biomaterial is a major event in determining the fate of the blood coagulation cascade. Platelet adhesion, aggregation, and coagulation are the primary responses when a foreign material comes in contact with blood [28]. The aggregated and star-shape-forming platelets in the prepolishing group reflected the same morphological index as for the activated platelet forms [29]. Nevertheless, these morphologies were markedly decreased in the postpolishing group (Fig. 4). Thus, the effect of E-polishing in preventing platelet adhesion on the metal surface may presumably endow the stent with an effective thromboresistant property. We hypothesized that nanopores formed by surface polishing could provide spaces for EVL incorporation. As expected, the amount of EVL in the nanoporous material surface was higher than that in the non-polished smooth surface (Fig. 6a). Meanwhile, the negative-pressure and lyophilization processes delayed the release of EVL from the surface (Fig. 6b). The efficacy of the drug present on the stainless-steel surface was examined by assessing the cellular responses to the drug. In this study, limitation of coronary stent represents in-stent restenosis (ISR), that is, the ingrowth of SMC to the lumen area of stent. The adhesive, migratory, and proliferative properties of SMC, leading to ISR, were all suppressed on the polished material (Figs. 5, 7, and 8). The interaction of cells with biomaterials is of key importance for the successful long-term implantation of medical devices. Furthermore, cell adhesion and spreading form the main parameters of cell biomaterial interaction [30]. These cellular responses are fundamental processes that are directly involved in pathological outcomes, such as wound healing, immune response, and biomaterial tissue integration [31, 32]. The indirect cellular response using Transwell inserts was performed to investigate whether the initial EVL released from the material surfaces within 1 day was because of “incorporation” or “wash-out.” After washout of the disk loaded with EVL, the SMC migration pattern was not significantly affected in the prepolishing group (Fig. 7), thereby implying that the drug released from the prepolishing surface before 1 day was simply laid on the surface and was not surface-incorporated.

## Conclusions

Although stainless steel has various advantages as a medical device, surface modification is required to exhibit effective cellular response and drug delivery. Therefore, in this study, micropores were formed on the surface of stainless steel through E-polishing to improve cellular response and maximize drug delivery efficacy. Platelet adhesion and SMC migration and proliferation, which are the primary events of thrombosis and ISR, were markedly inhibited by surface modification. Collectively, the results indicate that stainless steel can be used in various fields of biotransplantation by forming micropores. Moreover, the properties of the metal itself can be customized to medical devices.

## Data Availability

Not applicable.

## Abbreviations

EVL: Everolimus
mTOR: mammalian target of rapamycin
E-polishing: Electropolishing
SEM: Scanning electron microscopy
vSMC: vascular smooth muscle cell
PBS: Phosphate-buffered saline
AFM: Atomic force microscopy
Ra: Roughness average
PRP: Platelet-rich plasma
THF: *Tetrahydrofuran*
